# Efficacy of sigmoidoscopy for evaluating disease activity in patients with ulcerative colitis

**DOI:** 10.1186/s12876-022-02178-0

**Published:** 2022-02-27

**Authors:** Su Bum Park, Seong-Jung Kim, Jun Lee, Yoo Jin Lee, Dong Hoon Baek, Geom Seog Seo, Eun Soo Kim, Sang-Wook Kim, So Yeong Kim

**Affiliations:** 1 Department of Internal Medicine, Pusan National University School of Medicine, Pusan National Yangsan Hosptial, Yangsan, Republic of Korea; 2 Department of Internal Medicine, College of Medicine, Chosun University, 309, Pilmun-daero, Dong-gu, Gwangju, 61452 Republic of Korea; 3 Department of Internal Medicine, Keimyung University School of Medicine, Daegu, Republic of Korea; 4 Department of Internal Medicine, Pusan National University Hospital, Pusan National University School of Medicine, Busan, Republic of Korea; 5 Department of Internal Medicine and Digestive Disease Research Institute, Wonkwang University School of Medicine, Iksan, Republic of Korea; 6 Department of Internal Medicine, School of Medicine, Kyungpook National University, Daegu, Republic of Korea; 7 Department of Internal Medicine, Jeonbuk National University Medical School, Jeonju, Republic of Korea; 8 Department of Preventive Medicine, College of Medicine, Chosun University, Gwangju, Republic of Korea

**Keywords:** Ulcerative colitis, Sigmoidoscopy, Colonoscopy

## Abstract

**Background:**

Endoscopic assessment of disease activity is a key parameter in the management of ulcerative colitis. Whether sigmoidoscopy alone is sufficient to evaluate the disease activity in ulcerative colitis lacks studies.

**Methods:**

We retrospectively analyzed the medical records and endoscopic results of patients with ulcerative colitis followed by colonoscopy in seven tertiary hospitals between January 2012 and December 2018. Endoscopic disease activity was scored using the Mayo endoscopic subscore (MES) and Ulcerative Colitis Endoscopic Index of Severity (UCEIS) for each segment from the colonoscopy images. Concordance was evaluated by comparing the highest MES and UCEIS in the rectosigmoid and proximal regions to confirm the usefulness of sigmoidoscopy.

**Results:**

A total of 500 colonoscopic examinations from 333 patients were enrolled. Only in 7.6% [*k(kappa): 0.893, r(Spearman): 0.906, p* < *0.001*] and 8.6% [*k(kappa): 0.890, r(Spearman): 0.914; p* < *0.001*] of cases, MES and UCEIS scored more severely in the proximal colon. Comparison of active disease (MES ≥ 2) in the rectosigmoid area and the entire colon showed a high concordance rate [*k(kappa): 0.899, r(Spearman): 0.904, p* < *0.001*]. Endoscopic healing (MES = 0) also showed a high concordance rate [*k(kappa): 0.882, r(Spearman): 0.887, p* < *0.001*]. In 38 cases (7.6%) of patients with a higher MES in the proximal area, it was significantly higher in patients with previous extensive colitis.

**Conclusions:**

Sigmoidoscopy and colonoscopy showed a high concordance rate. Therefore, sigmoidoscopy is considered a sufficient substitute for colonoscopy. However, colonoscopy should be considered in patients with previous extensive colitis.

**Supplementary Information:**

The online version contains supplementary material available at 10.1186/s12876-022-02178-0.

## Background

Treatment strategies for ulcerative colitis are shifting from simple symptom control to complete remission of the disease itself [1, 2]. Endoscopic remission has been suggested as the main treatment goal to prevent permanent intestinal damage and disability [3, 4]. Recently, the Selecting Therapeutic Targets in Inflammatory Bowel Disease (STRIDE-II) consensus statements reported that endoscopic healing is a long-term target and that assessment can be achieved by sigmoidoscopy or colonoscopy [5]. The Mayo Endoscopic Subscore (MES) or Ulcerative Colitis Endoscopic Index of Severity (UCEIS) is most commonly used for the evaluation of endoscopic activity, and the most active site reflects the overall score [6, 7]. However, we often encounter more severe lesions in the proximal than the rectosigmoid area in clinical practice. Although most guidelines recommend sigmoidoscopy for endoscopic assessment because of the high risk of perforation in severe inflammation, there is no clear recommendation as to whether sigmoidoscopy or colonoscopy is recommended in most other cases. Two previous studies also reported completely different results [8, 9]. However, in both studies, important variables such as previous disease extent, severity, and rectal topical treatment were not reflected in the results. Therefore, it is questionable whether sigmoidoscopy can reflect the activity of the entire colon.

Therefore, this study aimed to analyze patients diagnosed with ulcerative colitis who underwent follow-up colonoscopy to determine whether sigmoidoscopy alone could reflect the degree of inflammation of the entire colon.

## Methods

### Patients

This retrospective multicenter study was conducted at seven tertiary academic hospitals in the southern region of South Korea. From January 2012 to December 2018, patients with ulcerative colitis whose disease activity was evaluated through colonoscopy were collected. Among patients who underwent colonoscopy, only those who had the cecum intubated were enrolled. Patients with failed cecal intubation, with a history of colorectal surgery, or those with unclear medical records or endoscopic data were excluded. A total of 500 endoscopic examinations in 333 patients were enrolled and analyzed. Patients' age, sex, duration of illness, disease severity, and treatment modalities were collected from medical records. In addition, the indications were confirmed through the endoscopy report and the results of biomarker such as fecal calprotectin and C-reactive protein performed during colonoscopy were collected.

### Endoscopic assessment for disease activity

Colonoscopy images stored for each segment were analyzed to evaluate the endoscopic disease activity. The MES and UCEIS were used to evaluate the endoscopic disease activity of ulcerative colitis. The MES ranges from 0 for a normal or inactive state to 3 for a severely active state [10]. The UCEIS has three subcategories: vascular pattern, bleeding, and erosion and ulcer, with a score of 0–2 for the vascular pattern and 0–3 for bleeding and erosion and ulcer, summing up to a total score of 0–8 [7]. For both MES and UCEIS, the highest score for each segment is reflected as the overall score.

Tertiary academic hospitals in South Korea must obtain the Accreditation of Qualified Endoscopy Unit hosted by the Korean Society of Gastrointestinal Endoscopy every 3 years. In the field of colonoscopy, the cecal intubation rate is an essential item, and the storage of colonoscopy images must contain at least eight high-resolution images depending on the segment, including the maximum intubation site image [11]. Therefore, despite being a retrospective study, we analyzed the concordance by obtaining MES and UCEIS for each segment based on relatively accurate data. Moreover, to lower interobserver errors, all authors from each tertiary center participating in the study communicated through a conference prior to data collection.

### Statistical analysis

Categorical data were analyzed using the frequency (%) analyses, χ^2^ test, and Fisher’s exact test, and quantitative data were analyzed using the independent sample t-test. For the agreement and correlation analysis of the two methods, sigmoidoscopy and colonoscopy, kappa coefficient, and Spearman’s correlation analysis were used. The kappa coefficients are classified as 0, poor; 0.01–0.20, slight; 0.21–0.40, fair; 0.41–0.60, moderate; 0.61–0.80, substantial; 0.81–1, nearly perfect. According to Landis and Koch, the Spearman correlation coefficient is distributed from −1 to +1, a value closer to −1 indicates a negative correlation, and a value closer to +1 indicates a positive correlation. Linear regression analysis was used for negative prediction values. Statistical significance was defined as *p*<0.05, and all statistical analyses were performed using SPSS Statistics version 26.0 (IBM Corp., Armonk, NY, USA).

## Results

### Patient’s basal characteristics

A total of 333 patients with ulcerative colitis underwent at least one colonoscopy to evaluate disease activity, and a total of 500 examinations were enrolled. The average age of the patients was 44 years, and 218 (65.5%) patients were males. Before colonoscopy, the extent of disease (n = 500) was proctitis in 206 cases (41.2%), left-sided colitis in 148 cases (29.6%), and extensive colitis in 146 cases (29.2%). There were 234 cases (46.8%) wherein topical 5-aminosalicylic acid (5-ASA) was used, and 70 cases (14.0%) wherein biologics were used. In 188 cases (37.6%), colonoscopy was performed to confirm disease activity in ulcerative colitis with flare-up. Colonoscopy was performed during hospitalization in 65 cases (13.0%) (Table 1).

**Table 1:** Baseline characteristics of 500 colonoscopic findings in 333 patients with ulcerative colitis

Patient characteristics	Number (%)
Total N	333 patients
Sex	
Male, n (%)	218 (65.5)
Female, n (%)	115 (35.5)
Mean Age (years, range)	44.13 ± 17.78 (17–80)
Total N	500 colonoscopic findings
Disease duration (month, range)	37.37 ± 48.88 (1–288)
Disease extent at last examination	
Proctitis (n, %)	206 (41.2)
Left-sided colitis (n, %)	148 (29.6)
Extensive colitis (n, %)	146 (29.2)
Treatment modality	
Topical 5-ASA	234 (46.8)
Oral 5-ASA	440 (88.0)
Glucocorticoid	125 (25.0)
Immunomodulator	141 (28.2)
Biologics	70 (14.0)
Indication for colonoscopy	
Response after initial diagnosis	103 (20.6)
Flare-up	188 (37.6)
Surveillance (No symptom)	209 (41.8)
Total Mayo score	3.57 ± 3.18(0–12)
Hospitalization	
Outpatient	453 (87.0)
Inpatient	65 (13.0)
Laboratory findings	
CRP (mg/dL)	1.46 ± 5.21 (0–55)
Fecal calprotectin (μg/g)	621.65 ± 777.18 (0–3409)

*5-ASA* 5-aminosalicylic acid, *CRP* C-reactive protein

### Agreement between sigmoidoscopy and colonoscopy

**Fig. 1:**
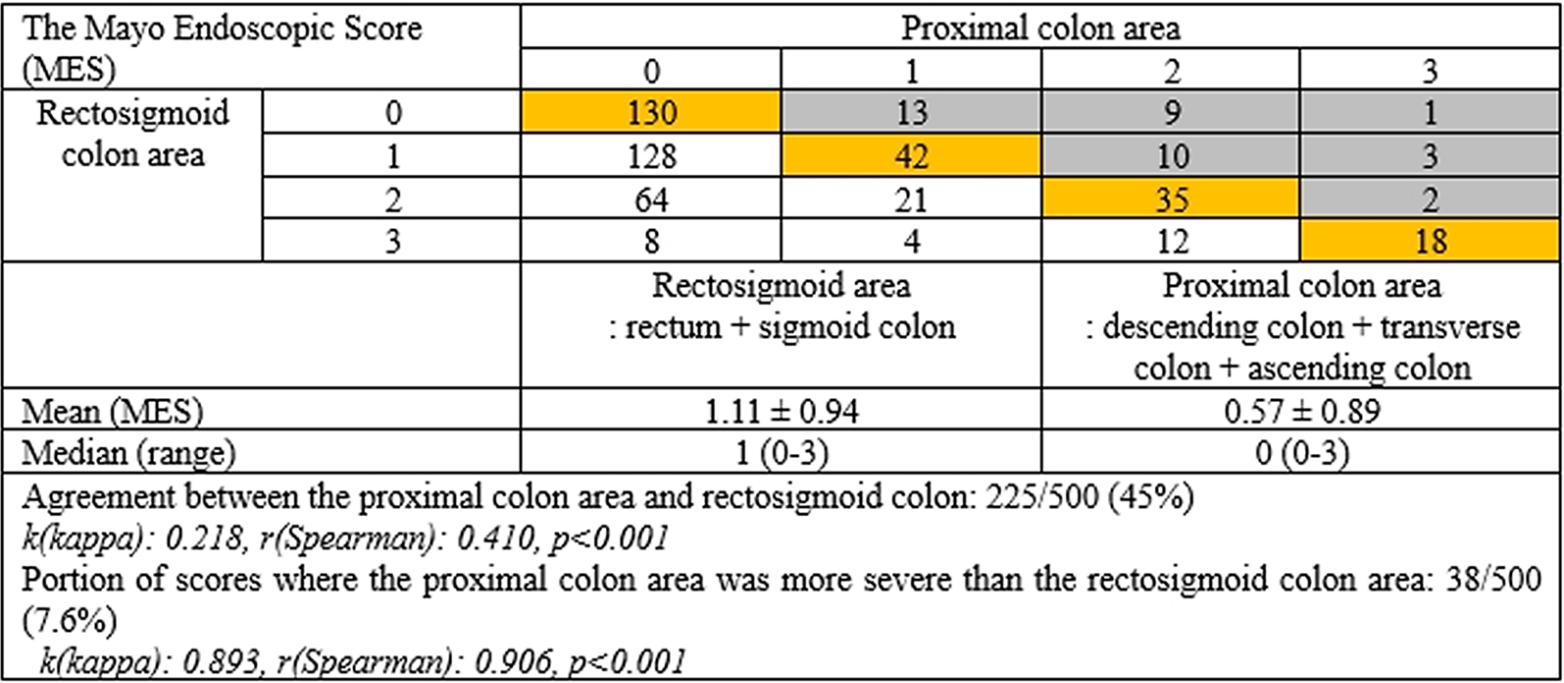
Analysis of the concordance between the proximal colon and rectosigmoid area: the Mayo Endoscopic Subscore

**Fig. 2:**
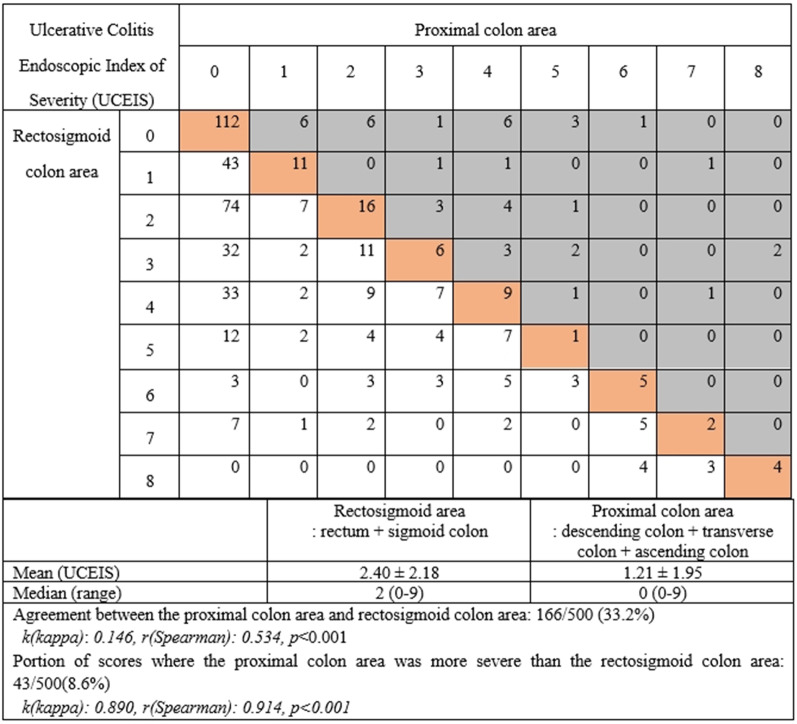
Analysis of the concordance between the proximal colon and rectosigmoid area: Ulcerative Colitis Endoscopic Index of Severity

Each segment of the colon was divided into two parts, a rectosigmoid area and a proximal colon area, depending on whether it could be examined by sigmoidoscopy. The disease activity of the rectosigmoid area was reflected in the highest score among the MES and UCEIS of the rectum and sigmoid colon, respectively. Similarly, the proximal colon area had the highest score among the descending, transverse, and ascending colon and cecum. The coincidence of the MES and UCEIS scores between the two areas was analyzed. Although the concordance between the two areas of the MES was as low as 45%, only 7.6% of the cases [*k(kappa): 0.893, r(Spearman): 0.906, p* < *0.001*] had a more severe proximal colon score (Fig. 1). Concordance was 33.2% in UCEIS, and the proportion of scores where the proximal colon area was more severe than the rectosigmoid colon area was 8.6%, confirming similar results [*k(kappa): 0.890, r(Spearman): 0.914, p* < *0.001*] (Fig. 2).

Sigmoidoscopy was defined as the highest score among MES and UCEIS for each segment in the rectosigmoid area, and colonoscopy was defined as the highest score in the entire colon to compare the usefulness of sigmoidoscopy and colonoscopy for endoscopic assessment of active disease and endoscopic healing. As an endoscopic evaluation for disease activity in patients with ulcerative colitis, MES ≥ 2 or UCEIS ≥ 5 is evaluated as active disease, and escalation of drugs should be considered [12, 13]. In MES, active disease confirmed by sigmoidoscopy and colonoscopy was consistent in 164 patients, inactive disease in 313 patients, and only 23 patients did not undergo sigmoidoscopy. There, sigmoidoscopy and colonoscopy showed high concordance rates [*k(kappa): 0.899, r(Spearman): 0.904, p* < *0.001*], and high concordance rates between sigmoidoscopy and colonoscopy were confirmed when classified by disease activity according to the UCEIS [*k(kappa): 0.918, r(Spearman): 0.921, p* < *0.001*] (Table 2). According to STRIDE-II, endoscopic healing should be measured as a long-term target. As per the definition, MES = 0 or UCEIS ≤ 1 is proposed compared with MES ≤ 1 to achieve a better disease outcome. Endoscopic healing (MES = 0) confirmed by sigmoidoscopy and colonoscopy were consistent in 130 patients, and only 23 patients did not undergo sigmoidoscopy. Sigmoidoscopy and colonoscopy showed high concordance rates [*k(kappa): 0.882, r(Spearman): 0.887, p* < *0.001*]. Endoscopic healing (UCEIS ≤ 1) also confirmed similar results [*k(kappa): 0.914, r(Spearman): 0.917, p* < *0.001*] (Table 3).

**Table 2:** Comparison of the concordance rates of active disease assessed by sigmoidoscopy and colonoscopy

Active disease (MES ≥ 2) N = 500		Colonoscopy		Active disease (UCEIS ≥ 5) N = 500		Colonoscopy	
		Yes	No			Yes	No
Sigmoidoscopy	Yes	164	0	Sigmoidoscopy	Yes	83	0
	No	23	313		No	12	405
k(kappa) = 0.899, r(spearman) = 0.904, *p* < 0.001,				k(kappa) = 0.918, r(spearman) = 0.921, *p* < 0.001			

*MES* Mayo endoscopic subscore, *UCEIS* Ulcerative Colitis Endoscopic Index of Severity

**Table 3:** Comparison of the concordance rates of endoscopic healing assessed by sigmoidoscopy and colonoscopy

Endoscopic healing (MES = 0) N = 500		Colonoscopy		Endoscopic healing (UCEIS ≤ 1) N = 500		Colonoscopy	
		Yes	No			Yes	No
Sigmoidoscopy	Yes	130	23	Sigmoidoscopy	Yes	172	20
	No	0	347		No	0	308
k(kappa) = 0.882, r(Spearman) = 0.887, *p* < *0.001*				k(kappa) = 0.914, r(Spearman) = 0.917, *p* < 0.001			

*MES* Mayo endoscopic subscore, *UCEIS* Ulcerative Colitis Endoscopic Index of Severity

### Which patients need colonoscopy to assess the disease activity?

In 38 cases (7.6%), a high endoscopic score was confirmed in the proximal area rather than in the sigmoidoscopy area in which colonoscopy was required for evaluating the activity. Therefore, we compared the cases in which disease activity could only be evaluated by sigmoidoscopy (n = 462) and the case in which colonoscopy was absolutely necessary (n = 38) (Table 4). We confirmed that disease extent is an important factor contributing to the need for colonoscopy (*p* < *0.001*), and in the case of extensive colitis, colonoscopy should be considered more actively. Moreover, colonoscopy was also required if the total Mayo score for ulcerative colitis disease activity was high (3.48 vs. 4.66, *p* = 0.02). There were no statistically significant differences in sex, age, disease duration, the indications for endoscopy, the use of topical 5-ASA, hospitalization, CRP or fecal calprotectin.

**Table 4:** Comparison of the cases wherein disease activity could be evaluated only with sigmoidoscopy and requiring colonoscopy

Patient characteristics	Sigmoidoscopy (n = 462)	Colonoscopy (n = 38)	*p* Value
Sex			0.784
Male, n (%)	306 (66.2)	26 (68.4)	
Female, n (%)	156(33.8)	12 (31.6)	
Mean age (year, range)	44.43 ± 18.07	39.79 ± 18.33	0.129
Disease duration (month, range)	37.72 ± 49.31	33.11 ± 43.73	0.576
Disease extent at last examination			< 0.001
Proctitis (n, %)	200 (43.3)	6 (2.9)	
Left-sided colitis (n, %)	138 (29.9)	10 (6.8)	
Extensive colitis (n, %)	124 (26.8)	22 (57.9)	
Indication for colonoscopy			0.831
Response after initial diagnosis	96 (20.8)	7 (18.4)	
Flare-up	172 (37.2)	16 (42.1)	
Surveillance (No symptom)	194 (42.0)	11 (28.9)	
Total Mayo score	3.48 ± 3.19	4.66 ± 2.93	0.022
Hospitalization			0.637
Outpatient	401 (86.8)	34 (89.5)	
Inpatient	61 (13.2)	4 (10.5)	
Use of topical 5-ASA			0.614
No	244 (52.8)	22 (57.9)	
Yes	218 (47.2)	16 (42.1)	
Laboratory findings			
CRP (mg/dL)	1.48 ± 5.33	1.16 ± 3.42	0.635
Fecal calprotectin (μg/g)	612.52 ± 775.52	805.50 ± 850.50	0.576

*5-ASA* 5-aminosalicylic acid, *CRP* C-reactive protein

## Discussion

In the present study, we confirmed high concordance of MES and UCEIS between the rectosigmoid area and the entire colon. In patients with ulcerative colitis, endoscopic assessment is a very important indicator for evaluating not only the severity of worsening symptoms but also mucosal healing as a long-term treatment target [14–16]. Our results suggest that sigmoidoscopy is sufficient as a follow-up test to evaluate disease activity after the diagnosis of ulcerative colitis.

The merits of sigmoidoscopy are that it is safe, cost-effective, has a short procedure time, and does not require sedation. However, there are concerns about whether sigmoidoscopy can represent the disease activity of the entire colon. First, atypical distributions such as rectal sparing and skipped lesions were identified in 12.6% of initial colonoscopies in patients with ulcerative colitis [17]. Second, more severe endoscopic findings were often found in the proximal region during follow-up colonoscopy after treatment in clinical practice. To date, there is no consensus on whether sigmoidoscopy alone can represent the disease activity of the entire colon. In the previous two studies, retrospective analysis of colonoscopy images of patients with ulcerative colitis confirmed the endoscopic evaluation of the rectosigmoid area, which can be confirmed by sigmoidoscopy and colonoscopy, and contradictory results were reported. According to Kato et al. [8] 27% (147/545) of patients with ulcerative colitis had maximum inflammation in the descending colon or proximal colon. They insisted that sigmoidoscopy was not sufficient for evaluating patients with ulcerative colitis and that colonoscopy would be necessary, especially in patients experiencing the first attack. However, this study could not determine the extent and severity of the previous disease. As mentioned above, atypical distribution at the time of diagnosis is relatively high; thus, there is a limitation in accurately reflecting it in the evaluation of disease activity. It also did not accurately reflect the definition of actual endoscopic healing (MES = 0 or UCEIS ≤ 1) or active disease (MES ≥ 2 or UCEIS ≥ 5). According to Colombel et al. [9] in only 3.7% (9/239) of cases the detection of active disease and 5.0% (7/139) of cases the assessment of endoscopic healing discordant findings were obsz 0erved between the rectosigmoid area and proximal area. They insisted on a high degree of correlation in the assessment of ulcerative colitis between sigmoidoscopy and colonoscopy. However, in this study, only patients undergoing induction treatment were enrolled in the etrolizumab phase 2 study; thus, it is challenging to represent all patients with ulcerative colitis. In addition, since this was a retrospective study and the video was analyzed, the boundary for each segment was ambiguous.

In contrast, our study is a multicenter study involving seven institutions, thereby minimizing patient selection bias, and it reviewed the extent and severity of ulcerative colitis at the time of diagnosis. We analyzed patients with more severe endoscopic findings in the proximal area and confirmed that colonoscopy was required for activity evaluation in patients with extensive colitis and high total Mayo score. In addition, although not statistically significant, fecal calprotectin was confirmed to be high in the group that required a colonoscopy. The reason fecal calprotectin is not statistically significant is thought to be that because it is a fecal examination, patients do not get tested in certain cases. Fecal calprotectin is a non-invasive biomarker that can predict disease activity in ulcerative colitis and has a high concordance with endoscopic findings [18–20]. Elevated fecal calprotectin levels suggest more severe inflammation, and colonoscopy may be necessary to confirm more proximal lesions. However, being a retrospective study, our study could not confirm the cut-off value for fecal calprotectin requiring colonoscopy. Therefore, future prospective studies are required.

Since suppository is a topical treatment, it is likely a risk factor for a mismatch between the proximal lesion and sigmoidoscopy. However, in our study, topical therapy did not affect the discrepancy between proximal and rectal colon lesions. This is probably because, in our study, colonoscopy was divided into fractions, and sigmoidoscopy not only examined the rectum but also included the sigmoid colon. In general, the principle of sigmoidoscopy is to check the region below the splenic flexus, that is, even the descending colon. We additionally analyzed the concordance between the left-sided colon (rectum, sigmoid, and descending colon) and the proximal colon (ascending and transverse colon). In only 2.4% of the cases [*k(kappa): 0.934, r(Spearman): 0.956, p* < *0.001*), the proximal colon had a more severe score (Additional file 1: Fig. 1). Therefore, when evaluating disease activity with sigmoidoscopy, a more accurate evaluation would be possible if the descending colon was intubated.

This study has certain limitations. First, this is a retrospective study of colonic images. The colonoscopy images analyzed in this study did not accurately represent the disease activity of the entire colon. However, this study was conducted at a tertiary university hospital in South Korea, which receives endoscopy certification every 3 years. Since all colonoscopies require storing high-resolution images for each segment, it is thought that more accurate data were enrolled. Second, only patients with ulcerative colitis who had undergone colonoscopy were enrolled in our study. Patients with severe inflammation who could not undergo colonoscopy were underestimated, and there was a relatively high probability of selection bias.

In conclusion, our study is the first multicenter study to show that sigmoidoscopy alone is sufficient to confirm disease activity. It is recommended to insert the endoscope up to the descending colon when performing sigmoidoscopy to increase the accuracy. In the case of patients with extensive colitis, colonoscopy should be considered as a test to confirm disease activity.

## Supplementary Information

**Additional file 1: Supplementary Figure 1.** Analysis of the concordance between the proximal colon and rectosigmoid area: the Mayo Endoscopic Subscore.

## Data Availability

The datasets analyzed during the current study and the full trial protocol are available from the corresponding author on reasonable request.

## Abbreviations

MES: Mayo endoscopic subscore
UCEIS: Ulcerative colitis endoscopic index of severity
STRIDE-II: Selecting therapeutic targets in inflammatory bowel disease
5-ASA: 5-Aminosalicylic acid; CRP, C-reactive protein
