# Supplementary material for: Efficacy of sigmoidoscopy for evaluating disease activity in patients with ulcerative colitis
Source: BMC Gastroenterol. 2022 Feb 27;22:83. doi: 10.1186/s12876-022-02178-0 (PMC8882296; doi:10.1186/s12876-022-02178-0)

**Supplementary Figure 1**. Analysis of the concordance between the proximal colon and rectosigmoid area: the Mayo Endoscopic Score.


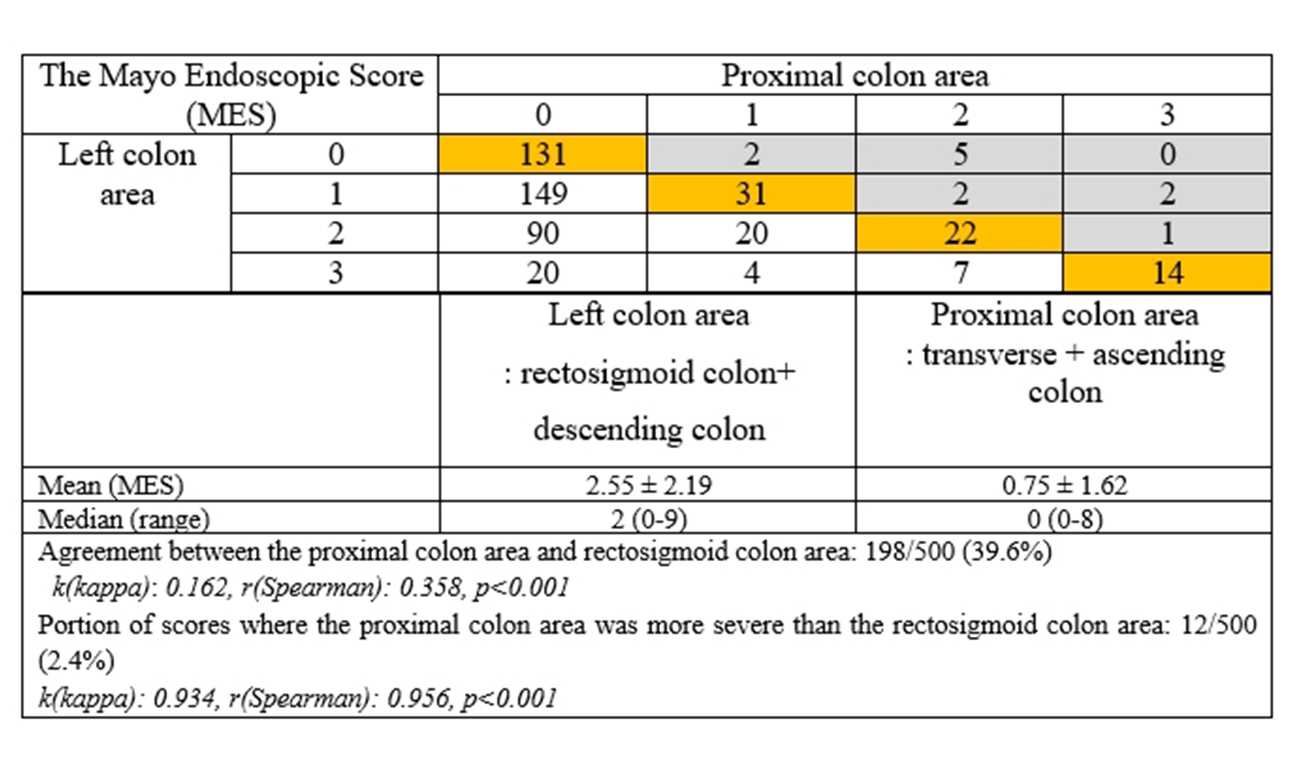

Supplement: Supplementary file 1 — Additional file 1: Supplementary Figure 1. Analysis of the concordance between the proximal colon and rectosigmoid area: the Mayo Endoscopic Subscore. [file 12876_2022_2178_MOESM1_ESM.docx]
